# Supplementary material for: Mapping the prevalence and covariates associated with home delivery in Bangladesh: A multilevel regression analysis
Source: PLoS One. 2024 Nov 12;19(11):e0313606. doi: 10.1371/journal.pone.0313606 (PMC11556713; doi:10.1371/journal.pone.0313606)
Supplement: Attachment — Submitted filename: Response to Reviewers.docx [file pone.0313606.s002.docx]

**Response to the Journal Requirements:**

When submitting your revision, we need you to address these additional requirements.

1. Please ensure that your manuscript meets PLOS ONE's style requirements, including those for file naming.

**Author’s Response:** We have prepared our manuscript after careful checking the submission guidelines that meets the PLOS ONE's style requirements.

2. Your ethics statement should only appear in the Methods section of your manuscript. If your ethics statement is written in any section besides the Methods, please move it to the Methods section and delete it from any other section. Please ensure that your ethics statement is included in your manuscript, as the ethics statement entered into the online submission form will not be published alongside your manuscript.

**Author’s Response:** We have replaced the ethics statement in the method section and removed from other section.

3. We note that Figure 2 in your submission contain map image which may be copyrighted. All PLOS content is published under the Creative Commons Attribution License (CC BY 4.0), which means that the manuscript, images, and Supporting Information files will be freely available online, and any third party is permitted to access, download, copy, distribute, and use these materials in any way, even commercially, with proper attribution. For these reasons, we cannot publish previously copyrighted maps or satellite images created using proprietary data, such as Google software (Google Maps, Street View, and Earth). For more information, see our copyright guidelines: <http://journals.plos.org/plosone/s/licenses-and-copyright>.

We require you to either (1) present written permission from the copyright holder to publish these figures specifically under the CC BY 4.0 license, or (2) remove the figures from your submission:

**Author’s Response:** We didn’t copy the map from other sources. We generated the map using ArcGIS software that we clearly mentioned in the method section. For creating the map, we used the data (prevalence of home delivery) from MICS 2019 and the Bangladesh shape file from a freely available online source: <https://data.humdata.org/dataset/cod-ab-bgd>. We have added a statement in the figure caption following previous papers in PLOS journals as below:

*“*We generated this map using data obtained from the MICS 2019 survey available on: “[*https://mics.unicef.org/surveys*](https://mics.unicef.org/surveys)*”*, and then we used the base shape file of Bangladesh from freely available online source: [*https://data.humdata.org/dataset/cod-ab-bgd*](https://data.humdata.org/dataset/cod-ab-bgd)*?”*

**Data availability statement:**

**We have uploaded the data files “Home delivery_Data set” and shape file “BD Shape File” for Bangladesh as Supporting Information Zip file.** We hope that this time, the data-sharing statement will be alright to go for the further process.

4. Please review your reference list to ensure that it is complete and correct. If you have cited papers that have been retracted, please include the rationale for doing so in the manuscript text, or remove these references and replace them with relevant current references. Any changes to the reference list should be mentioned in the rebuttal letter that accompanies your revised manuscript. If you need to cite a retracted article, indicate the article’s retracted status in the References list and also include a citation and full reference for the retraction notice.

**Author’s Response:** We have checked all the references we used. We didn’t cite any retracted article. We have added some recent references (18-24) and removed some old references. Please see the reference section.

**Response to the Reviewers' comments:**

**Reviewer #1**:

**Comment**: Dear Authors,
I hope this email finds you well. I wanted to let you know that I found your article to be well-written, covering an interesting and helpful topic that could prove valuable for future recommendations and actions. I strongly recommend that the article be published with no significant changes. However, I would like to suggest updating the reference list, as approximately 40% of the sources listed are dated before 2015. Thank you for taking the time to consider my feedback.

**Author’s Response:** Dear reviewer, thank you for your appreciation and time to review our paper. We agree with your argument that almost 40% of references are dated before 2015. For your kind information, we found a few references that are available on the home delivery, rather most of the recent works focused mainly on the institutional delivery. However, we have updated some references this time according to your suggestion and also incorporated some recent references that worked on home delivery. Please check the reference section.

Besides that, we had to keep some references dated even before 2010 due to supporting some arguments in our problem statement and discuss some results of our study. We found those references are crucial to rationalize our statements. So, we hope you will also agree with us; however, if you suggest some references that are strongly required to improve our paper, we will be happy to include them.

**Reviewer #2:**

**Comment**: Line 100: skilled “attendance” is it a typo error of attendant.

**Author’s Response:** Dear reviewer, thank you for your time to review our paper. We corrected the typo you pointed out.

**Comment**: Line 132: ever-married: what is the meaning of it?

**Author’s Response:** We have defined the term “ever-married” with reference as follows:

*“The ever-married women were those who had been married at least once in their lives though they may not be currently married [26]*”

**Comment**: Line 131: and study procedure can be found elsewhere, it should be replaced with proper place as author is given reference for it.

**Author’s Response:** We have replaced it with the report of MICS 2019. Now it reads:

*“The detailed information on sampling technique, questionnaire, and study procedure can be found in the MICS 2019 report [25]*”

**Comment**: Line 148: wealth status (poorest, poorer, middle, richer, richest). Representation doesn’t look here, it can be replaced with income range, like low income (0–100 USD per week/month, 100–500 USD ) so on.

**Author’s Response:** Thank you for your comment. MICS measures the household wealth status based on ownership of different household assets. In Bangladesh MICS 2019, 25 (groups of) variables that were used for the construction of the Bangladesh Wealth Index. **The wealth index is assumed to capture the underlying long-term wealth through information on the household assets and is intended to produce a ranking of households by wealth, from poorest to richest.** The wealth index does not provide information on absolute poverty, current income or expenditure levels. Each household in the total sample is then assigned a wealth score based on the assets owned by that household and on the final factor scores obtained as described above. The survey household population is then ranked according to the wealth score of the household they are living in and is finally divided into 5 equal parts (quintiles) from lowest (poorest) to highest (richest). In MICS report, there is a separate variable for wealth status that we used where the quintiles of wealth status were named as poorest, second, middle, fourth, and richest. We also replaced “poorer” with “second”, and “richer” with “fourth” in this study (please see in the method section). The aforementioned description are taken from the MICS 2019 report. There is no estimation of the income amount (like in USD) available in the MICS data sets. Previous studies also used this variable as wealth quintile. Please see a previous study and MICS report 2019 below:

Bangladesh Bureau of Statistics (BBS) and UNICEF Bangladesh. Progotir Pathey, Bangladesh Multiple Indicator Cluster Survey 2019, Survey Findings Report. Dhaka, Bangladesh: Bangladesh Bureau of Statistics (BBS); 2019.

Chowdhury TR, Chakrabarty S, Rakib M, Winn S, Bennie J. Risk factors for child stunting in Bangladesh: an analysis using MICS 2019 data. Arch Public Heal. 2022;80:1–12.

We also added a statement with reference in the method section in this study as follows:

*“The household wealth status was calculated based on the ownership of different household assets using principal component analysis (PCA) [28].”*

**Comment**: Line 149: exposure to media . What is the role of exposure of media in this study?

**Author’s Response:** The Bangladesh MICS, 2019 collected information on exposure to mass media, where the information was collected on exposure to newspapers / magazines, radio and television among women 15-49 years. We categorized women having exposure to media if they had access to either reading newspapers / magazines, or listening radio or watching television following the guidelines of MICS and previous literature (please see ref below).

Saleheen AAS, Afrin S, Kabir S, Habib MJ, Zinnia MA, Hossain MI, et al. Sociodemographic factors and early marriage among women in Bangladesh, Ghana and Iraq: An illustration from Multiple Indicator Cluster Survey. Heliyon. 2021;7.

We added the following statement in the method section now:

*“We classified women as having exposure to media if they had access to read newspapers or magazines, listen to the radio, or watch television.”*

In response to your question- “what is the role of exposure of media in this study”, we included this variable based on the previous studies where media exposure was found to be associated with the place of delivery. Though we didn’t find any significant association for the individual level exposure to media, a significant association of community level media exposure was significantly associated with the home delivery in this study, and we discussed this finding.

**Comment**: Classification should be modified or add reference where from it was adopted words like “Poorest” and “Richest”, in my concern it should be replaced with the range of income.

**Author’s Response:** We have provided the references of MICS 2019 report, where the wealth status also categorized as quintile (poorest, second, middle, fourth, richest). Please see ref 25.

**Comment**: Line 189: Define this line “9,166 (weighted) women who had at least one live delivery in the two years before to 190 the survey was part of this investigation.”

**Author’s Response:** We have rewritten the line as follows:

*“A total of 9,166 (weighted) women who had at least one live delivery in the two years preceding the survey were included in this study.*”

**Comment**: While shading some light of the data given in the Table 1 section Age at first marriage/union, ≤18 years, and number of correspondents is 6632. As 7069 correspondents were of 20–34 -year range. As well as 6632 were less than 18 year. Please explain it, I am confused now.

**Author’s Response:** These are two distinct variables. The variable “Age of women” represents the current age of women at the time of survey, where we found 7069 women were from 20-34 years age group. On the contrary, the variable “Age at first marriage/union” provides the information on about how old the woman was at the time of first marriage / union? Hence, the variable “Age at first marriage/union” provides the prior information, not current, and consequently, it shows that 7069 respondents were of 20–34 -year range, but 6632 were less than 18 year at their first marriage / union.

**Comment**: Line 292 and line 295 is seems the repetition of the same statement in a different way. Rewrite it please.

**Author’s Response:** Thank you for pointing this out. We have removed the 2^nd^ line.

**Comment**: Line 286 and line 300 also looks similar, better this sentence should be used in the later part of discussions to make it general statement with combining the effect of education and wealth status.

**Author’s Response:** Thank you for this comment. We have placed this sentence at the end of the paragraph discussing association between socioeconomic status and home delivery in a single statement with combining the effect of education and wealth status. Now it reads:

*“Additionally, women from higher socioeconomic group with higher education and wealth status may be more empowered to make decisions for themselves, obtain information, and be financially independent enough to support themselves, travel to a medical facility and pay for services when needed, as well as to easily absorb health-related messages from the media and from medical professionals [46,47]*”

**Comment**: Check the spellings and reference throughout use correct format, as ref 14, 16 and 22. Rest if possible to support study some recent references also can be added.

**Author’s Response:** We followed the PLOS style for referencing. We have added some recent references also. Please see refs 18 to 24.
