# Supplementary material for: Mapping the prevalence and covariates associated with home delivery in Bangladesh: A multilevel regression analysis
Source: PLoS One. 2024 Nov 12;19(11):e0313606. doi: 10.1371/journal.pone.0313606 (PMC11556713; doi:10.1371/journal.pone.0313606)
Supplement: Attachment — Submitted filename: Comments PLOS ARTICLE.docx [file pone.0313606.s003.docx]

Comments

Generally, this paper is essential to maternal and child health. The authors dealt with the phenomenon with a clear methodological step. I congratulate them on that. It’s great work, congratulations to the authors. However, they should pay attention to these comments and work on them to improve the paper.

Lines 1 and 2, the title reads: “Mapping the prevalence and Covariates Associated with Home Delivery in Bangladesh: A Multilevel Regression Analysis”. For consistency’s sake, they could consider capitalizing “prevalence” as the major keywords started with capital letters.

Line 41 to 43, they started the sentence with “though”. Technically, Abstract should be written in abstracting terms. As such, authors could consider removing “though”, and “however” from the abstract.

In line 43, the authors stated that “However, reasons behind this need to be explored in community-level”. the use of “reasons” appears as if the authors are going to present real reasons. Meanwhile, the work is quantitative. Quantitative language should be used here instead of qualitative terms/words. Preferably, they can use terms such as “predictors”, “plausible factors”, “determinants” and others. “Reasons” sounds more in a qualitative sense.

Line 51, the authors stated that “The overall weighted prevalence of home delivery was 46.41%”. it is appropriate to add the confidence intervals for this overall prevalence.

Line 51-58, the authors only presented fixed effects results, it will be appropriate if they capture the random effects results as well here, briefly.

In line 68, the authors included “delivery care” as a keyword and I wonder how that can be a keyword in this study.

In lines 76-77, the authors stated that “In most, but not all, nations during the past few decades, there has been a marked decline in home births”. This is a good point though; however, I was expecting a recent citation to align with this statement. However, the authors cited 1985, 1997. This is several decades ago as we are in 2024.

Lines 78-80, Reference 11 and 12 are citations in 2006 and 2012 on Maternal mortality trends. This is less acceptable as recent estimates of maternal mortality are available for use. Authors should consider revising references that are too old in the entire background section.

In line 108, the authors stated “Bangladesh has implemented several initiatives to address maternal health……..”. However, they didn’t elaborate on these initiatives. The section will read better and scientifically good if the authors narrate/synthesize the strategies/initiatives, adopted in Bangladesh, the success made by such strategies, and the failures if any. They have to do such a comparative analysis.

In lines 108 to 111, the authors presented “Issues such as inadequate healthcare infrastructure, geographical barriers, limited access to skilled birth attendants, and socioeconomic disparities continue to affect maternal health outcomes”. These are probable factors that might have been identified by earlier studies so to avoid plagiarism, authors have to reference this statement accordingly.

Lines 111 to 113, reads “Our study’s findings help in identifying the obstacles to health facility delivery and the variables influencing maternal fatalities during in-home birth in Bangladesh”. Is this a justification or statement of the problem? Authors should come clearly and state the problem and the justification for the study separately. They should not combine the problem statement and justification.

Authors should present the study design first before talking about the data source in the methods section.

In lines 136 to 142, the authors didn’t talk about “others” in the responses. Were there “others” in the responses given by the respondents and yes, how are “others” managed in this study?

Line 148, ANC visits were classified as no visits, 1-3 visits, and 4 or above visits. What informs this classification? Currently, the practice is less than 8 visits as poor and 8 or more as desirable. It could be appropriate if they reclassify ANC according to current standards. A similar thing applies to the community ANC variable. Here, authors classified them into less than 50% having 4 or more ANC visits which is even not consistent with their earlier ANC classification at the individual level. they should reconcile this.

Line 149, the authors should consider explaining how they generated exposure to mass media as the dataset used does not have a variable called mass media, to the best of my knowledge.

Line 249, the authors should check this grammar “was to mapping” and correct it appropriately, “The main objective of this study **was to mapping** the prevalence of home delivery practice”.

In the policy implication section, authors have to be specific. Who or which agency or ministry should implement what? They should direct their policy implications to specific agencies or ministries/departments of Bangladesh. For instance, targeted efforts by the Ministry of Health, Reproductive Unit or Public Health Division of Bangladesh should focus on……

Authors should consider discussing policy implications after the conclusion. So they should shift policy implication to the last section after the conclusion.

Finally, authors should check for grammatical errors and if any exist, correct them. Thank you.
