# Supplementary material for: Mapping the prevalence and covariates associated with home delivery in Bangladesh: A multilevel regression analysis
Source: PLoS One. 2024 Nov 12;19(11):e0313606. doi: 10.1371/journal.pone.0313606 (PMC11556713; doi:10.1371/journal.pone.0313606)
Supplement: Attachment — Submitted filename: Response to reviewers.docx [file pone.0313606.s004.docx]

**Response to the Reviewers' comments:**

**Comment:** Generally, this paper is essential to maternal and child health. The authors dealt with the phenomenon with a clear methodological step. I congratulate them on that. It’s great work, congratulations to the authors. However, they should pay attention to these comments and work on them to improve the paper.

**Author’s response:** Dear Reviewer, Thank you for the time and for considering our paper for reviewing. We appreciate your effort and are thankful for your appreciation and valuable feedback.

**Comment:** Lines 1 and 2, the title reads: “Mapping the prevalence and Covariates Associated with Home Delivery in Bangladesh: A Multilevel Regression Analysis”. For consistency’s sake, they could consider capitalizing “prevalence” as the major keywords started with capital letters.

**Author’s response:** Thank you for pointing this out. We have capitalized it in the title.

**Comment:** Line 41 to 43, they started the sentence with “though”. Technically, Abstract should be written in abstracting terms. As such, authors could consider removing “though”, and “however” from the abstract.

**Author’s response:** We have revised the introduction section of the abstract and removed ‘though’ and ‘however’ from the abstract. Now it reads:

*Bangladesh has made an intense effort to improve maternal healthcare facilities including facility delivery, but the number of home deliveries is still very high Therefore, this study aims to find out district-wise prevalence and determine the individual and community-level covariates related to home delivery among women in Bangladesh.*

**Comment:** In line 43, the authors stated that “However, reasons behind this need to be explored in community-level”. the use of “reasons” appears as if the authors are going to present real reasons. Meanwhile, the work is quantitative. Quantitative language should be used here instead of qualitative terms/words. Preferably, they can use terms such as “predictors”, “plausible factors”, “determinants” and others. “Reasons” sounds more in a qualitative sense.

**Author’s response:** Thank you for this insightful comment. We have changed the sentence and revised the section. Please see the previous response.

**Comment:** Line 51, the authors stated that “The overall weighted prevalence of home delivery was 46.41%”. it is appropriate to add the confidence intervals for this overall prevalence.

**Author’s response:** Thank you for your suggestion. We have added the confidence intervals for the prevalence in the abstract.

**Comment:** Line 51-58, the authors only presented fixed effects results, it will be appropriate if they capture the random effects results as well here, briefly.

**Author’s response:** We have added a statement from the random effects results in the abstract. Please see the statement below:

*The intercept-only regression model demonstrates that the likelihood of women from various clusters having home delivery varied significantly (variance: 1.47, standard error [SE]: 0.117), indicating the applicability of multilevel regression modeling.*

**Comment:** In line 68, the authors included “delivery care” as a keyword and I wonder how that can be a keyword in this study.

**Author’s response:** We have removed ‘delivery care’ from the keywords.

**Comment:** In lines 76-77, the authors stated that “In most, but not all, nations during the past few decades, there has been a marked decline in home births”. This is a good point though; however, I was expecting a recent citation to align with this statement. However, the authors cited 1985, 1997. This is several decades ago as we are in 2024.

**Author’s response:** We have updated these with some recent references (Please check refs 6-8).

**Comment:** Lines 78-80, Reference 11 and 12 are citations in 2006 and 2012 on Maternal mortality trends. This is less acceptable as recent estimates of maternal mortality are available for use. Authors should consider revising references that are too old in the entire background section.

**Author’s response:** Dear reviewer, Thank you for your observation. We have tried to update the references for the entire background section. The references you are referring to, was to support the following statement *“The expansion of institutional delivery coverage and the use of skilled birth attendants during deliveries are only a couple of the measures that have been put forth to lower this maternal, fetal, and newborn mortality.”* To support the maternal mortality rate and trends, we used several recent references those are mostly after 2019. Please check the refs 11-14.

**Comment:** In line 108, the authors stated “Bangladesh has implemented several initiatives to address maternal health……..”. However, they didn’t elaborate on these initiatives. The section will read better and scientifically good if the authors narrate/synthesize the strategies/initiatives, adopted in Bangladesh, the success made by such strategies, and the failures if any. They have to do such a comparative analysis.

**Author’s response:** We have added some statements demonstrating the available health services and initiatives of Bangladesh with references. Now it reads:

*Bangladesh has implemented several initiatives related to maternal health services to address maternal health. The key maternal health services available in Bangladesh include antennal care, post-abortion care, basic essential obstetric care (EOC), comprehensive EOC, postnatal care for mothers, intensive care unit for mothers, introduction of health vouchers scheme for poor women, deployment of community-based skilled birth attendants, and introduction of the midwifery programme [28–30]. But not all health facilities, particularly those in rural regions in Bangladesh, offer all kinds of maternity and newborn health services [29]. Consequently, despite progress, Bangladesh still faces challenges in reducing maternal mortality [31].*

**Comment:** In lines 108 to 111, the authors presented “Issues such as inadequate healthcare infrastructure, geographical barriers, limited access to skilled birth attendants, and socioeconomic disparities continue to affect maternal health outcomes”. These are probable factors that might have been identified by earlier studies so to avoid plagiarism, authors have to reference this statement accordingly.

**Author’s response:** We already added the references for this statement. Please see refs 31 & 32.

**Comment:** Lines 111 to 113, reads “Our study’s findings help in identifying the obstacles to health facility delivery and the variables influencing maternal fatalities during in-home birth in Bangladesh”. Is this a justification or statement of the problem? Authors should come clearly and state the problem and the justification for the study separately. They should not combine the problem statement and justification.

**Author’s response:** We have revised the sentence now and focused on the objective of the study. We have separated the problem statement and justification of the study now.

**Comment:** Authors should present the study design first before talking about the data source in the methods section.

**Author’s response:** We have re-organized the section, keeping study design before the data source in the method section as per reviewers comment.

**Comment:** In lines 136 to 142, the authors didn’t talk about “others” in the responses. Were there “others” in the responses given by the respondents and yes, how are “others” managed in this study?

**Author’s response:** We have clarified about the other’s home now. In the question, there were two options for home delivery along with the name of some other institutions. The two options for home delivery were “respondent’s home” and “other’s home”, where the other’s home meant the home of neighbours, friends, relatives or any birth attendants, and we have clarified this in the method section now as follows:

*The other’s home referred to the home of neighbors, friends or relatives of the respondents, or any birth attendant’s home.*

**Comment:** Line 148, ANC visits were classified as no visits, 1-3 visits, and 4 or above visits. What informs this classification? Currently, the practice is less than 8 visits as poor and 8 or more as desirable. It could be appropriate if they reclassify ANC according to current standards. A similar thing applies to the community ANC variable. Here, authors classified them into less than 50% having 4 or more ANC visits which is even not consistent with their earlier ANC classification at the individual level. they should reconcile this.

**Author’s response:** Thank you for your insightful comment. In response, we have recategorized the individual-level ANC visits into four categories: no visits, 1-3 visits, 4-7 visits, and 8 or more visits. This reclassification aligns with the current WHO recommendations for antenatal care (ANC) visits. Following this adjustment, we have reanalyzed the data accordingly.

Regarding the community-level ANC visit variable, we encountered a significant limitation. When we attempted to create a separate community-level variable based on the criterion of 8 or more visits as indicative of high community-level ANC, we observed that less than 1% of participants fell into this category. This extremely low prevalence rendered the variable ineffective for meaningful analysis and interpretation.

Consequently, we have excluded the community-level ANC visit variable from our study because of low percentage (less than 1%) in one category of two. We appreciate your feedback, which has been instrumental in refining our analysis. Please refer to the revised tables for the updated results.

**Comment:** Line 149, the authors should consider explaining how they generated exposure to mass media as the dataset used does not have a variable called mass media, to the best of my knowledge.

**Author’s response:** We have clarified how we generated the media exposure variable. Now it reads:

*Media exposure was created as a dichotomous variable. Women were categorized as having media exposure if they reported access to at least one of the following information sources like reading newspapers/magazines, listening radio, or watching television. Those with access to any of these sources were classified as ‘yes’ for media exposure, while those without access to any were classified as ‘no’ [38].*

**Comment:** Line 249, the authors should check the grammar “was to mapping” and correct it appropriately, “The main objective of this study **was to mapping** the prevalence of home delivery practice”.

**Author’s response:** Thank you for pointing this out. We have corrected this and fixed the grammatical errors throughout the manuscript.

**Comment:** In the policy implication section, authors have to be specific. Who or which agency or ministry should implement what? They should direct their policy implications to specific agencies or ministries/departments of Bangladesh. For instance, targeted efforts by the Ministry of Health, Reproductive Unit or Public Health Division of Bangladesh should focus on……

**Author’s response:** Thank you for this insightful comment. We have revised the recommendations and now it reads:

*The study's conclusions may have a significant impact on interventions and policy decisions that are proposed for specific agencies and ministries in Bangladesh to lower the prevalence of home birth in Bangladesh. The Directorate General of Health Services (DGHS) under the Ministry of Health and Family Welfare along with different non-government organizations should spearhead awareness campaigns on the benefits of skilled birth attendance and the risks of home delivery. Concurrently, the Ministry of Women and Children Affairs, in collaboration with the Ministry of Education, should focus on improving education and awareness among women regarding the benefits of skilled birth attendance and the potential risks associated with home delivery. Additionally, interventions should address socioeconomic barriers by providing financial support for transportation and improving the affordability of maternal health services. The DGHS, Bangladesh should also enhance the availability and quality of healthcare facilities in rural areas in order to curve the reliance on home delivery in these regions.*

**Comment:** Authors should consider discussing policy implications after the conclusion. So they should shift policy implication to the last section after the conclusion.

**Author’s response:** We have shifted the policy implication to the last section after the conclusion.

**Comment:** Finally, authors should check for grammatical errors and if any exist, correct them. Thank you.

**Author’s response:** We have thoroughly checked and fixed the grammatical errors throughout the manuscript this time.
